# Acerola (*Malpighia emarginata*) Anti-Inflammatory Activity—A Review

**DOI:** 10.3390/ijms25042089

**Published:** 2024-02-08

**Authors:** Remigiusz Olędzki, Joanna Harasym

**Affiliations:** 1 Department of Biotechnology and Food Analysis, Wroclaw University of Economics and Business, Komandorska 118/120, 53-345 Wroclaw, Poland; remigiusz.oledzki@ue.wroc.pl; 2 Adaptive Food Systems Accelerator-Science Centre, Wroclaw University of Economics and Business, Komandorska 118/120, 53-345 Wroclaw, Poland

**Keywords:** malpighia, acerola, nutritional prophylaxis, bioactive substances, antioxidants

## Abstract

The manuscript provides an overview of recent scientific reports on the properties and range of health-promoting effects of acerola (*Malpighia emarginata* DC) fruits and leaves. Acerola is a natural raw material that, in its unprocessed form, is known to be a rich source of vitamin C and polyphenolic compounds. For this reason, the consumption of acerola may provide a number of health-promoting benefits, particularly related to its strong anti-free radical effects. The review discusses anti-inflammatory and anticancer effects of acerola fruit and leaves as well as its therapeutic effects on selected physiological processes in the human system. Their biochemical mechanisms are also explained. Recommendations for the consumption of acerola in the prevention of inflammatory and free radical diseases are presented. The part of the article devoted to anticancer effects of acerola describes the possibilities of using the edible parts of this raw material to obtain products and preparations of potential use in cancer prevention and therapy.

## 1. Introduction

A holistic approach to health is gaining popularity among consumers, which is reflected in the search for more effective natural treatments including choosing the right ingredients and diet. Malpighia fruits (*Malpighiae emarginata DC*/*Malpighia glabra* L.), commonly known as acerola, Guarani cherry, Barbados cherry or wild crepe myrtle, come from a plant originating in Yucantan. It then spread among the Americas till Brazil, and then around the equator. The plant presents interesting and valuable health-promoting properties. Increasingly recognized in the market of exotic vegetable raw materials, acerola is beginning to be widely appreciated by consumers, mainly as a fruit with a high content of vitamin C (ascorbic acid), as well as polyphenolic compounds (e.g., anthocyanins) and carotenoids [1]. Previous reports on acerola present the fruit (pomace, seeds and juice) and leaves as a raw material rich in carotenoids (e.g., lutein, β-carotene), polyphenolic compounds (hydroxycinnamic acids, flavonoids), pheophytin and chlorophyll derivatives, which can act as ingredients for potential natural nutraceuticals [2].

As access to this commodity in Western European markets is becoming easier, the plant is becoming an increasingly common purchase target for consumers. On the consumer market, acerola can be purchased in various forms, mainly in the form of preparations, as an additive in food (e.g., in yogurts), in dietary supplements, and in the form of fresh fruit (mainly available in large supermarkets and specialty health food stores). Acerola is rapidly gaining popularity in nutrition and food technology not only because of its valuable taste qualities, but also because of its unique health-promoting properties. Due to the fact that in recent years the results of many interesting scientific studies on this unique plant have been published, it has become an important goal of this article to organize this information for usefulness in nutrition education.

The aim of this article is to summarize the recent reports concerning acerola as a potent source of bioactive compounds, especially those of anti-inflammatory activity. The manuscript presents the characteristics of botanical origin and habitat of acerola, biochemical properties of this fruit, its antioxidant and anti-inflammatory effects, as well as the impact of acerola consumption on the composition of the gut microbiome, gastro- and hepatoprotective and hepato-regenerative effects of acerola and the anti-cancer activity.

## 2. Botanical Characteristics and Habitat

The pomegranate-leaf malpighia is native to the warm regions of Central America, and its territorial range includes southern areas of North America (Texas, New Mexico, Lower California, Sonora in Mexico), Central America, semi-northern areas of South America and the Caribbean islands [3]. Acerola grows in river valleys in warm and dry tropical forests characterized by high fertility, such as in the Balsas River Valley and the central valleys of Oaxaca, Mexico [4]. Brazil is the world’s largest producer, consumer and exporter of acerola, with eighteen varieties of acerola registered, according to the Brazilian Ministry of Agriculture, Livestock and Supply, among which the most popular are BRS 235-Apodi, BRS 236-Cereja and BRS 237-Roxinha [5].

Under natural conditions (original habitats), acerola takes the form of an undersized evergreen tree with oval green leaves. In its regions of origin, acerola reaches 2–3 m in height and is often cultivated as a hedge plant and at the same time as a fruit tree, which develops red, drupe-like fruits with yellow flesh, similar in shape and size to the fruit of the common cherry (*Prunuscerasus* L.) or the bird cherry (wild cherry, sweet cherry) (*Prunusavium* L.) [6,7]. The most valuable part of acerola is its fruit, about 1.5–2.0 cm in diameter, which can be eaten raw, subjected to drying and can be processed to prepare decoctions, juices or jams [7].

## 3. Biochemical Properties of Acerola

The fruit of acerola, due to the existence of many botanical varieties, presents a wide range of biochemical properties, which is the result of the high genetic variability of this plant (the presence of a large number of genotypes within the species) and the high phenotypic variability of individual traits of this plant. The results of the study confirm that acerola is primarily a valuable source of ascorbic acid and malic acid. Ascorbic acid is the main organic acid of acerola, the concentration of which (in the fruit pulp, depending on the variety) shows values ranging from 1.18 g to 2.43 g*100 g^−1^ fresh weight, making acerola as valuable a source of ascorbic acid as, for example, the fruit of the black currant (*Ribesnigrum* L.) (1.5–3.0 g*100 g^−1^ fresh weight). For this reason, consumption of acerola can strongly stimulate the immune system, including by increasing the number and activity of immune cells, such as lymphocytes. Biochemical analysis of acerola fruit (pomace) also revealed a high β-carotene content of 5.84 mg*g^−1^ dry weight, making this raw material potentially as valuable a source of β-carotene as, for example, the common carrot (*Daucuscarota* L.) [5].

Depending on the variety, acerola has been shown to have a high total content of polyphenolic compounds, ranging from 378.69 to 444.05 mg GAE (gallic acid equivalent) * 100 g^−1^ dry weight, which determines that this fruit can be described as one of the richest dietary sources of polyphenols, on par with, for example, the fruit of black chokeberry (*Aronia melanocarpa*) [5]. It has been confirmed that the phenolic compound with the highest concentration in acerola pomace is naringenin (at 478 µg*g^−1^ dry weight), or 5,7,4′-trihydroxyflavanone), which has been attributed with anti-atherosclerotic and estrogen-like effects [8]. The main anthocyanin compound in acerola fruit was found to be cyanidin-3-rhamnoside, which is present at 149–682 µg*g^−1^ fresh weight. Phenolic compounds such as quercetin-3-glucoside, isorhamnetin, catechin, procyanidin A2, hesperidin, chlorogenic acid and trans-resveratrol were also found in acerola fruit [5].

Research has shown that the content of ascorbic acid in acerola fruit decreases during the ripening process [1]. As a result, the amount of ascorbic acid in unripe fruit is much higher than that of polyphenolic compounds and carotenoids. Moreover, studies have confirmed that during the ripening of acerola fruit, the concentration of kaempferol, luteolin, rutoside, chlorogenic acid and apigenin increases [9]. However, the content of p-coumaric acid and ferulic acid is significantly reduced during the ripening process [10].

The presence of the aforementioned compounds in acerola means that consumption of the edible parts of this plant (e.g., the fruit) can have beneficial effects on a number of physiological and biochemical processes in the human body [5]. The main groups of bioactive compounds in acerola fruit are presented in Table 1.

## 4. Antioxidant and Anti-Inflammatory Effects of Acerola

**Figure 1:**
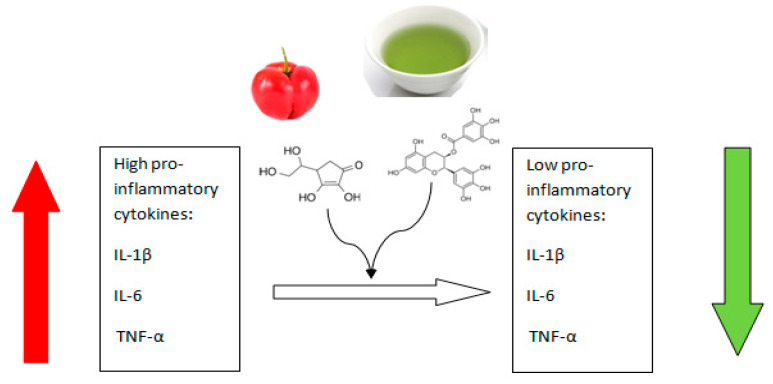
Mechanism of synergistic interaction between ascorbic acid (contained in acerola) and epigalato-catechin-3-gallate (contained in green tea) in reducing the secretion of pro-inflammatory cytokines. Red arrow means increase, green arrow means decrease. Own diagram based on the research [13].

It has been observed that the antioxidant and anti-inflammatory effects of this raw material on the human body are particularly noticeable when acerola is consumed with other products with high bioactive potential, such as green tea (*Camellia sinensis* L.). It has been shown that the bioactive substances contained in both plants, when taken simultaneously and for a long time, can have a therapeutic effect on many pathophysiological processes in a synergistic manner. Functional products (blends) derived from both acerola and green tea exhibit high antioxidant and anti-inflammatory properties. The bioactive compounds contained in both plants can reduce oxidative stress in macrophages, which occurs during the inflammatory response. Adjunctive synergism between ascorbic acid (contained in high amounts in acerola) and epigallato-catechin 3-gallate (contained in high amounts in green tea) is responsible for the protective effects of the described mixture of both raw materials [13]. The physiological and cellular effect of this synergistic interaction is to modulate the inflammatory response by reducing the secretion of pro-inflammatory cytokines such as IL-1β, IL-6 and TNF-α- Figure 1. Therefore, it is recommended to consume both of these products (green tea and acerola) as part of the prevention of chronic inflammatory diseases, such as atherosclerosis, diabetes, irritable bowel syndrome and arthritis, among others [13].

Table 2 shows the types of health-promoting effects of bioactive substances contained in acerola fruit.

However, it should be noted that in the presented study, the ingredients used to obtain the tested product (mixture) were combined in only one ratio: 80% fresh acerola juice and 20% aqueous green tea extract (*v*/*v*). The presented experiment did not analyze the in vitro effects on the inflammatory response in cells of mixtures with a different ratio of the two ingredients [13]. In addition, it should also be taken into account that the maltodextrin used in the experiment to produce a functional product represents an additional source of energy (in the form of carbohydrates) for the body and thus may contribute to an increase in glucose intake, resulting in an increase of the amount of reactive oxygen species produced during the metabolism of these carbohydrates in the body.

In the body, glucose is converted to sorbitol, which, hitting the polyols pathway, leads to the production of reactive oxygen species (ROS). Glucose in the form of glucosamine-6P (D-glucosamine-6-phosphate) is converted to hexosamine pathway resulting also in the formation of O-GlcNAc-proteins (O-glycosylated proteins) and increased production of TGF-β (transforming growth factor β1) [21]. Studies on cell lines have shown that TGF-β1 may be one of the factors responsible for the formation of pancreatic cancer metastasis to the liver and contribute to local tumor progression. At the same time, TGF-β1 factor can stimulate unwanted fibrous tissue development in tumors, as a result of which connective tissue (so-called stroma) comes into contact with tumor cells (desmoplasia phenomenon) [22]. Thus, the maltodextrin used in the experiment in question to produce a functional product can have a significant impact on the resultant antioxidant potential.

**Figure 2:**
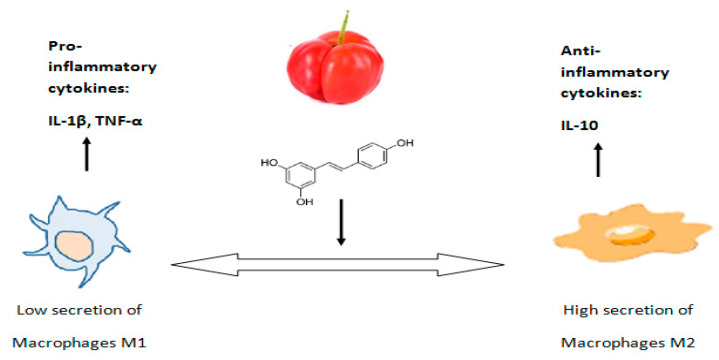
Mechanism of the effect of polyphenolic compounds contained in acerola fruit pulp on reducing secretions by M1 macrophages and increasing secretions by M2 macrophages in the colon and liver. Own diagram based on the research [20,23].

In animal studies, it was shown that in rats given a high-fat diet enriched with acerola fruit pulp, there was a reduction in the number of M1 macrophages in the colon and liver [20]. M1 macrophages react with Toll-like receptor 4 and produce pro-inflammatory cytokines such as TNF-α and IL-1β, causing low to moderate inflammation in the tissue. The aforementioned study also showed that animals on a high-fat diet enriched with acerola fruit pulp had a concomitant increase in the number of M2 macrophages in the colon and liver (compared to animals on a high-fat diet without acerola fruit pulp) [20]. Also, the appearance of M2 macrophages was associated with the secretion of an anti-inflammatory cytokine such as IL-10 by these cells [20] (Figure 2).

It is suggested that the anti-inflammatory effects of acerola are mainly due to the polyphenolic compounds contained in this fruit, which, depending on the variety, can range from 1296.4 mg GAE*100 g^−1^ to 1606.8 mg GAE*100 g^−1^ [11,24].

The limitation of this study is the use of only male animals (rats) which belonged to only one age group (30-day-old animals). The interesting input, especially now, when the Western population is aging, would be the elder organisms. The elderly, in whose bodies there is an impairment of many regulatory processes and a decrease in the integration of cells and organs, also differently respond to supplementation. This results in an inability to maintain homeostasis under physiological stress conditions in older organisms. As a result of the body’s reduced ability to maintain homeostasis, significant pharmacokinetic and pharmacodynamic changes occur in older organisms, that increase or decrease the body’s sensitivity to bioactive substances [25].

Other studies indicate that total polyphenolic compounds in acerola fruit pulp are found in the range as high as from 2418 mg*100 g^−1^ to 2844 mg*100 g^−1^ [26]. Polyphenolic compounds of acerola with specific anti-inflammatory properties are gallic acid and flavonols such as quercetin and myricetin [23,27]. Meanwhile, the total flavonoid content of acerola fruit can range from 35.56 to 39.97 mg EC (quercetin equivalent) * 100 g^−1^ g [11]. Based on other studies, the total flavonoid content of acerola fruit pulp has been confirmed to range from 479 mg*100 g^−1^ to 542 mg*100 g^−1^ [26]. Additionally, other polyphenolic compounds such as, hesperidin, kaempferol, caffeic acid, caftaric acid, chlorogenic acid, procyanidin B1, procyanidin A2, trans-resveratrol and epicatechin have also been found in acerola fruit, which may exhibit anti-inflammatory and antioxidant activity [11]. The anthocyanin content of the fruit pulp has been confirmed at about 2.7 mg cyanidin-3-glucoside * 100 g^−1^ of fresh raw material, while that of squeezed acerola juice is at 46.9–52.3 mg cyanidin-3-glucoside * L^−1^ of fresh product [28].

The other group of bioactive compounds with anti-inflammatory properties present in acerola are carotenoids, such as β-carotene [29]. The carotenoid content of different varieties of acerola depends largely on the period in which these fruits are harvested (in the dry or rainy season) and has been confirmed to range from 9.4–40.6 µg of β-carotene equivalent * 1 g^−1^ of raw material [30]. Among the carotenoids present in acerola, compounds such as lutein, β-cryptoxanthin and α-carotene have also been confirmed [31]. The total amount of vitamin A (beta-carotene) in acerola juice corresponds to an amount of about 760 UI of vitamin A * 100 mL^−1^ of product [32].

The anti-inflammatory properties of acerola may also depend on the large amounts of ascorbic acid it contains, which exhibits strong antioxidant activity [29]. It has been shown that the amount of vitamin C in acerola juice, corresponds to an amount of 1760 mg of vitamin C * 100 mL^−1^ of product [32]. Analyses of the acerola fruit indicate that the raw material contains ascorbic acid in the range of 1500–4500 mg*100 g^−1^, which is an amount about 50–100 times higher than the ascorbic acid content of oranges and lemons [33]. In contrast, another study found that the ascorbic acid content of acerola fruit pulp ranges from 3742.66 mg*100 g^−1^ to 5073.38 mg*100 g^−1^ of product [26]. In a study conducted by Righetto, it was shown that the antioxidant activity of acerola juices significantly depends on the synergistic effect of the components of different fractions, mainly ascorbic acid and polyphenolic compounds [28].

It is indicated that acerola fruits, which are characterized by particularly high vitamin C content, and at the same time, a high content of polyphenolic compounds, come from crops established primarily in Caribbean countries such as Barbados, Trinidad and Tobago and Haiti. In these regions, the fruit reaches a maximum intense red color at full maturity, accompanied by a high content of anthocyanin polyphenolic compounds in the skin [34].

It has been suggested that carotenoids may exert anti-inflammatory effects by modulating the composition of the intestinal microflora [35]. It has previously been shown that a high-fat diet causes changes in the composition of intestinal bacteria, involving a decrease in *Bifidobacterium* spp. and *Lactobacillus* spp. and an increase in *Enterobacteriaceae*, *Escherichia coli* and *Enterococcus* spp. [36]. In contrast, rats that were fed a high-fat diet enriched with an acerola fruit preparation showed increased activity of *Bifidobacterium* spp. and *Lactobacillus* spp. in the gastrointestinal tract and a reduction in *Enterobacteriaceae*, *Escherichia coli* and *Enterococcus* spp. [37].

Also, studies conducted on adults with obesity showed that the population of Lactobacillus spp. (especially the *L. acidophilus* species) is increased by consuming a fermented soy beverage that was enriched with acerola fruit. This effect was not observed when the test subjects were given the fermented soy beverage without the addition of acerola. However, the aforementioned product did not exert a preventive effect (neither maintain nor cause growth) in the population of *Lactobacillus* spp. (*L. acidophilus* species) when given to subjects with a normal weight-to-growth ratio [18].

However, it should be noted that the group of normal-weight subjects studied included only five volunteers (two men and three women) in a narrow age range (aged 20 to 33) and with an average body mass index (BMI) of 21.69 kg/m^2^. In contrast, the obese study group consisted of as many as thirteen volunteers (six men and seven women) in a significantly wider age range (aged 31 to 67) with an average BMI of 33.20 kg/m^2^ [18].

In another study on the effects of acerola, it was shown that consumption of this raw material can significantly reduce the content of the pro-inflammatory cytokine TNF-α, or tumor necrosis factor, in the liver, in a situation of damaging (toxic) effects of, for example, carbon tetrachloride on the liver [16]. In an experiment, the protective effect of acerola was demonstrated when rats were subjected to the toxic effects of carbon tetrachloride (CCl_4_). It was found that when animals exposed to the toxic effects of carbon tetrachloride were given *Malpighia glabra* L. leaf extract, there was a significant (up to 42%) reduction in the amount of TNF-α (pro-inflammatory cytokine) in the liver compared to animals treated with CCl_4_ who did not receive *Malpighia glabra* L. leaf extract [16]. It was also confirmed that the therapeutic result obtained was 3.5 more favorable than that obtained in animals treated with CCl_4_ and simultaneously treated with silymarin. It has been shown that treatment of animals poisoned with carbon tetrachloride with silymarin (i.e., a mixture of three flavones—silybin, silydiamine and silycristine) which is commonly used after liver damage caused by drugs or toxic substances such as ethanol or methanol) results in a reduction of TNF-α in the liver by only 12% [16].

In the experiment presented here, the acerola extract was administered orally to the test animals in doses in a fairly large range, from 1 g/kg to 5 g/kg of body weight. One has to wonder whether there would be differences in the results obtained if a comparison was made between the body’s response of the test animals to the minimum, as well as to the maximum dose of acerola extract [16].

In other animal studies, where rats were given acerola (*Malpighia emarginata* DC/*Malpighia glabra* L.) leaf extract at three doses; 200 mg*kg^−1^, 400 mg*kg^−1^ and 800 mg*kg^−1^, a reduction in serum liver enzymes such as alanine aminotransferase (ALT) by 13, 14 and 26%, respectively, and a reduction in aspartate aminotransferase (AST) activity by 21, 21 and 24%, respectively, was observed. In the described experiment, a significant 102% increase in serum catalase activity was also confirmed as a result of animals taking acerola leaf extract. In addition, it was proven that all amounts of acerola leaf extract administered to the animals induced a greater reduction in serum TNF-α levels compared to animals that received only silymarin. It has been suggested that bioactive compounds detected in the leaves of *Malpighia emarginata* L., such as coumarins (capensin, daphnoretin and scopoletin), flavonoids (mainly quercetin and apigenin glycosides) and phenolic acids (mainly cinnamic acid and quinic acid derivatives) may be responsible for the confirmed anti-inflammatory and hepatoprotective properties of acerola [16].

In a study using another model organism for toxicology and drug interactions, the striped danio (*Danio rerio*), it was shown that acerola (*Malpighia emarginata* DC/*Malpighia glabra* L.) seeds can exhibit potent anti-inflammatory effects that are triggered by the presence of irritants in foods. In addition, acerola’s pain-relieving effects have been proven [38]. It was confirmed that acerola, especially when administered to these animals together with graviola (*Annona muricata* L.) showed an inhibitory effect on acute nociception and inflammation in the abdominal cavity (abdominal inflammation) in adult striped danio (*Danio rerio*) caused by irritants such as formalin, capsaicin and cinnamaldehyde [38]. It has been confirmed that acerola has an attenuating effect on receptor (nociceptive) pain, resulting from mechanical or chemical irritation of sensory nerve receptors, caused, for example, by inflammatory mediators such as cytokines, histamine or arachidonic acid products [39]. It has been suggested that the polyphenolic substances in acerola interact through the nitrergic system. It has previously been shown that nitrergic neurons synthesize nitric oxide (NO), which, when released in the central nervous system, causes a reduction in blood pressure and a concomitant decrease in sympathetic nervous system activity, manifests as a reduction in pain intensity [40]. In addition, it is indicated that substances contained in acerola may participate in the activation of membrane guanylate cyclase (pGC), which catalyze the synthesis reactions of the secondary signal transmitter cyclic GMP (cGMP) [38]. The accumulation of cyclic cGMP results in a decrease in the intracellular concentration of calcium ions (Ca^2+^), which in turn leads to smooth muscle cell relaxation and subsequent pain reduction [38,41].

The Dias study showed that consumption of acerola juice in obese mice can prevent further weight gain and dyslipidemia when the test animals were fed a hypercaloric diet (the so-called cafeteria diet (4.12 kcal/g)), which increases body fat in a short period of time. Consumption of acerola juice has been confirmed to lower triglyceride levels while reducing inflammation in adipose tissue expressed by a reduced IL-10/TNF-α ratio [15].

The described effects were probably due to the effects of polyphenolic compounds present in acerola juice [15].

In the described study, the animals to which the hypercaloric diet was applied received food administered by gavage. However, it should be noted that the test animals, in addition to the forced diet, simultaneously had free access to water and standard food throughout the experiment [15]. Thus, the caloric intake of the test animals may have varied and, for some of the test animals, may have been higher than their daily caloric requirements.

**Figure 3:**
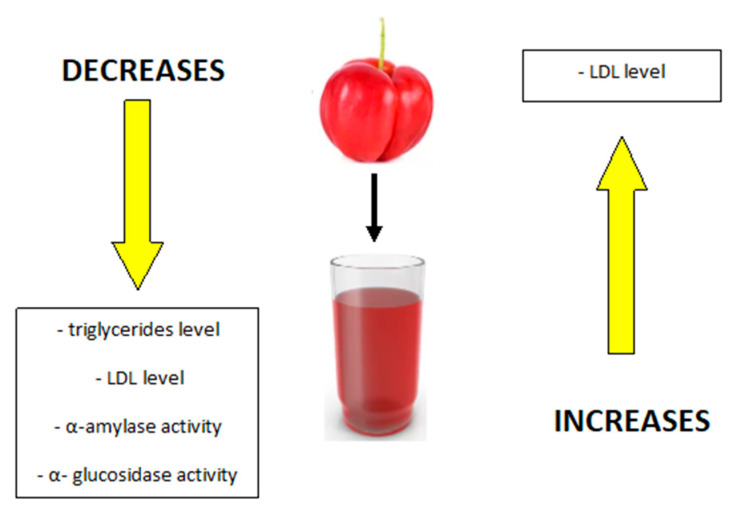
The effect of acerola juice consumption on the level of triglycerides, lipoproteins and the activity of amylolytic enzymes. Own diagram based on the research [15].

It has also been confirmed that the extract from acerola pomace can inhibit digestive enzymes such as α-amylase and α- glucosidase and thus may aid in the prevention or treatment of obesity—Figure 3. It has been suggested that consumption of acerola pomace extract may also reduce the development of diseases that coexist with obesity, such as type 2 diabetes [42]. These beneficial effects are explained by polyphenolic compounds such as catechin, epicatechin gallate, epicatechin, siringic acid, p-coumaric acid and quercetin present in acerola pomace extract [42].

It has also been shown that the fruit of acerola (*Malpighia emarginata* DC.), sourced from cultivation in the central region of Cuba, can exhibit effective protection against oxidative damage for human skin fibroblasts. It has been confirmed that polyphenolic compounds such as cyanidin 3-O-rhamnoside and pelargonidin 3-O-rhamnoside, caffeic acid hexoside, dihydrocavoylquinic acid, coumaroyl hexoside, and flavonols such as the glycosylated forms of quercetin and kemferol present in acerola can reduce fibroblast apoptosis induced by intracellular oxidative stress. The aforementioned polyphenolic substances also reduce the oxidation of lipids and proteins and significantly increase the activity of antioxidant enzymes such as catalase and superoxide dismutase [43].

Studies indicate that polyphenolic substances contained in the fruit of acerola (*Malpighia emarginata* DC.) can also reduce inflammatory processes in the intestines by restoring intestinal bacterial flora and inhibiting the growth and action of pathogens. This reduces the production of endotoxins produced by pathogenic microorganisms [44]. An additional property and anti-inflammatory effect of polyphenolic compounds from acerola is the inhibition of activated M1 macrophages and the inhibition of the production of pro-inflammatory mediators such as the interleukin, IL-6 [15].

Research results suggest that eating acerola fruit may be effective in reducing weight and obesity. It was shown that animals that were fed a high-fat diet with added acerola had a lower body fat content calculated on the basis of subcutaneous and retroperitoneal fat content. Thus, it is indicated that these animals were at a lower risk of obesity and weight gain in a high-fat diet situation [45].

Animal studies have provided the additional observation that the use of a high-fat diet that was simultaneously enriched with acerola fruit results in increased catalase activity [45]. Arguably, this has to do with the body’s adaptation to remove reactive oxygen species (hydrogen peroxide) that accompany animal organisms on a high-fat diet. In the situation described, increased catalase activity may be induced as a response of the Nrf2 (nuclear factor erythroid 2-related factor 2) transcription factor to oxidative stimuli in the form of increased amounts of reactive oxygen species generated by a fat-rich diet [46,47].

The observations presented should be treated with caution, since the results obtained were based on an experiment involving only male animals (rats). Moreover, in the experiment conducted, the animals were fed a high-fat diet for 7 weeks before receiving the acerola preparation. For this reason, the results presented here do not provide knowledge on the effect of acerola as a preventive treatment against obesity and inflammatory processes in animals that were fed a standard diet (which was characterized by a percentage of nutrients at: 12% fat, 28% protein and 60% carbohydrates and an energy value of 3.24 kcal/g) [20].

## 5. Effects of Acerola on the Composition of the Gut Microbiome

The changes in people consuming fermented soy beverage with acerola were also accompanied by beneficial changes in the gut microbiome. It was shown that the number of commensal *Firmicutes* and *Bacteroidetes* decreased in normal-weight subjects (with normal BMI) who consumed fermented soy beverage with acerola. On the other hand, consumption of acerola in the form of the described synbiotic (acerola in combination with probiotic bacteria *Lactobacillus* and *Bifidobacterium*) by subjects of normal weight (normal BMI) resulted in a more than 4.5-fold increase in the number of *Actinobacteria*, which were mainly represented by highly beneficial microorganisms of the genus *Bifidobacterium*. Also, in subjects of abnormal body weight (with abnormal BMI), consumption of fermented soy beverage with acerola caused similar beneficial changes. The consequence of consuming (in both healthy and overweight individuals) fermented soy beverage with added acerola is the development of beneficial bacteria, such as *Bifidobacterium* and *Prevotella*, accompanied by a decrease in the release of pro-inflammatory cytokines such as IFN-γ, TNF-α and IL-12. A co-linear positive consequence is a reduction in the population of *Clostridiaceae* bacteria, which in turn are credited with being involved in initiating the production of pro-inflammatory cytokines such as TNF-α and IL-1β [18].

In addition, the use of acerola as a fruit additive, and thus prebiotic, to fermented soy beverage has been shown to increase the viability and resistance of probiotic bacteria to gastrointestinal conditions and stimulate the growth of probiotic bacteria such as *Bifidobacterium* spp. (especially *Bifidobacterium longum* BB-46) and *Lactobacillus* in the colon [18].

Consumption of acerola fruit has also been shown to reduce the overgrowth of bacteria such as *Enterococcus*, *Clostridium cocoides* and *Eubacterium rectall*, whose endotoxins are responsible for activating the transcription factor NF-κΒ, a key trigger for cell proliferation in the colonic epithelium. Therefore, in order to reduce the risk of colon cancer, it is recommended to supplement the diet with products containing acerola fruit [37,48].

Consumption of acerola fruits in the presence of probiotic bacteria can separate additional benefits related to microbial biotransformation of polyphenols contained in this raw material. It has been shown that polyphenols contained in acerola fruit can be biotransformed by *Lactobacillus acidophilus* La-5 and *Lacticaseibacillus casei* 01, resulting in increased bioavailability and higher antioxidant activity of these substances. In addition, it has been shown that acerola fruit purees enriched with probiotic cultures as a result of fermentation processes are characterized by increased lactic acid (8.45–15.44 mg*mL^−1^), acetic acid (0.05–1.05 mg*mL^−1^) and reduced glucose and fructose content compared to acerola fruit purees without the addition of probiotic cultures [49].

It was also confirmed that acerola fruit purees with the addition of probiotic cultures have higher antioxidant activity, as a result of the conversion of oligomeric and polymeric polyphenolic substances (such as proanthocyanidins) into monomeric substances (such as catechins and epicatechins), which have higher antioxidant properties than the original substances [45].

Similarly, favorable effects on the antioxidant activities of acerola and the bioavailability of the bioactive compounds it contains have been demonstrated for probiotic isolates of *Lactobacillus casei* L-26, *Lactobacillus fermentum* 56, *Lactobacillus paracasei* 106 and *Lactobacillus plantarum* 53 [11].

The results presented here were obtained from an experiment that was only an in vitro simulation of water and electrolyte absorption and fermentation processes in the colon. Thus, the in vitro model of the large intestine used, does not fully reflect the actual microbiological processes to which unabsorbed and undigested carbohydrate substances are subjected to in the lower section of the human gastrointestinal tract (large intestine).

## 6. Gastro Protective Effects

It has been shown that polysaccharides extracted from acerola (from the peels and pulp) may have gastroprotective effects. The results of structural analysis indicated that the carbohydrate fraction extracted from acerola by-products is a heteropolysaccharide (pectic in nature) with a complex structure, containing glucose, arabinose, galactose, D-galacturonic acid and arabinogalactan molecules. The analyzed fraction of heteropolysaccharides can have a stimulating effect on the renewal process of intestinal epithelial cells, whose functional functions have been impaired due to infiltration of the dermis by inflammatory cells. On the basis of in vivo experiments, it has been shown that polysaccharides extracted from acerola fruit can reduce oxidative stress in the gastrointestinal mucosa. Consumption of polysaccharides from acerola also reduces (by 56.5%) the content of dialdehydumalonic acid (MDA), accumulated in gastric tissues due to ethanol consumption [19].

It has been observed that a reduction in the level of lipid peroxidation of cell membranes in gastrointestinal tissues under oxidative stress (e.g., ethanol-induced) occurs due to an increase in the antioxidant potential of these tissues, resulting from an increase in the concentration of glutathione (GSH—γ-glutamylcysteinylglycine)—a potent endogenous antioxidant synthesized in animals. It is assumed that the polysaccharides in acerola increase the activity of glutathione reductase and γ-glutamylcysteine synthetase, which are responsible for increasing the concentration of reduced glutathione (also by reducing GSSH, or oxidized glutathione). Therefore, the increased level of reduced glutathione (GSH) in the epithelial tissue of the stomach and intestines as a result of acerola consumption, confers effective protection of these organs against damage caused by radical-forming agents and substances (such as ethanol). At the same time, it was shown that the polysaccharide fractions of acerola not only do not show cytotoxic effects against the epithelial cells of the stomach and intestines, but also stimulate the process of their regeneration. Based on the results of the described studies, the use of polysaccharides extracted from acerola is postulated for the production of preparations that have a protective and regenerative effect on the gastric mucosa [19].

## 7. Hepatoprotective and Hepato-Regenerative Effects

Acerola leaves have been shown to be a raw material that is also a rich source of secondary metabolites that have high hepatoprotective potential. In vivo studies have confirmed that acerola leaf extract exhibits protective effects against liver cells. This conclusion was based on the observation that consumption of acerola leaf extract reduces in the blood the elevated activities of alanine aminotransferase (ALT), aspartate aminotransferase (AST, AspAT) and tumor necrosis factor α (TNF-α, cachectin), under conditions of exposure of this organ to toxic agents. At the same time, it was observed that acerola leaf extract is able to significantly (more than 20 times) increase in the blood the activity of catalase, whose function is to break down hydrogen peroxide (to water and oxygen), which is formed from superoxide radicals formed under oxidative stress. It is indicated that bioactive substances from groups such as coumarins (capensin, daphnoretin and scopoletin) present in acerola leaves may be responsible for this co-protective and hepatoregenerative effect, flavonoids (mainly glycosides such as quercetin and apigenin), phenolic acids (cinnamic acid and quinic acid derivatives), and amino acids such as homoisoleucine and phenylalanine [16].

Consumption of acerola leaf extract also lowers TNF-α levels in the blood, indicating its strong anti-inflammatory potential, which is comparable to the anti-inflammatory properties of silymarin (a complex of flavonolignans: Silibinin and its isomers -isosylicin, silicristin and silydianin), extracted from the seed husks of the spotted thistle (*Silybum marianum*) and which is used in the treatment of toxic liver damage and cholestatic diseases [16].

It is worth noting that in the experiment presented here, the test animals were exposed to a hepatotoxic agent (carbon tetrachloride) after a 14-day intake of the acerola leaf preparation, when the tissues of these animals were highly saturated with bioactive compounds. In the experiment in question, no studies were performed that took into account the hepatoprotective and regenerative effects of acerola against the organisms of animals that had previously been subjected to toxic effects of xenobiotics on the liver before taking the acerola preparation.

## 8. Impact on Detoxification of the System

In addition to the fruit, acerola leaves are also used to make nutritional or supplement products. They have been shown to be a prolific source of vitamin C, which is present at 34 mg*100 g^−1^, and glucuronic acid (33.04% of the total organic acid content), which has a particularly important function in the body due to its high detoxification properties. Glucuronic acid via glucuronyltransferase (UDP) can combine with endogenous toxic substances, converting them into water-soluble glucuronates, which are removed from the body by the kidneys. In addition, D-glucuronic acid molecules, together with N-acetylglucosamine, are substrates for the construction of hyaluronic acid, an important polysaccharide in connective tissue, which, through its water-binding property, imparts adequate elasticity to the dermis [16].

## 9. Stimulating Effect on the Synthesis of Steroid Hormones (Hormone-Forming Effect)

The presence of β-sitosterol, which, like cholesterol, may be the starting compound for the synthesis of androgens (steroid sex hormones), has also been detected in acerola leaves. Consumption of β-sitosterol can increase the concentration of steroid sex hormones (e.g., testosterone) in the body, thus contributing to the stimulation of processes related to the expansion of muscle mass. Therefore, β-sitosterol-containing acerola extract can be used as a safe alternative to unhealthy and often illegal (included in the list of banned substances according to the World Anti-Doping Agency—WADA), synthetic performance-enhancing substances (such as anabolic steroids) [50].

It has also been confirmed that the leaves of *Malpighia glabra* Linn. cultivated in Egypt, due to their high content of β-sitosterol and flavonoids, exhibit strong cytotoxic properties. Therefore, consuming preparations (extracts) of *Malpighia glabra* Linn. leaves can inhibit the growth of breast and colon cancer, as well as reduce the risk of stomach cancer [50,51].

## 10. Anticancer Activity

**Figure 4:**
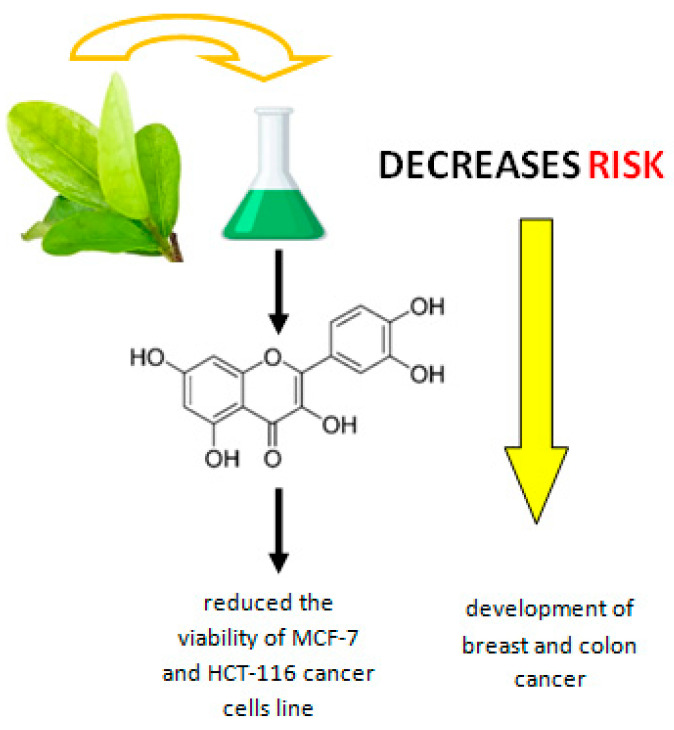
The process of inhibiting the viability of breast cancer cells (MCF-7 cell line) and colon cancer cells (HCT-116 cell line) by polyphenolic substances contained in acerola leaves. Own diagram based on the research [50,52].

It has been shown that substances in acerola leaves, such as tetranorditerpenes named acerolanin A, B and C (tetranorditerpenoids with a rare 2H-benz[e]inden-2-one substructure) can reduce the viability of breast (MCF-7 cell line) and colon (HCT-116 cell line) cancer cells, see Figure 4. For this reason, acerola leaf extracts may be an important part of nutritional prevention for people at high risk of developing breast cancer, i.e., those with identified mutations in the BRCA1 and BRCA2 genes, or those with a family history of breast cancer [52]. Substances contained in the fruit of acerola are also attributed to anticancer properties.

The presented results allow us to conclude that the tetranorditerpene substances contained in acerola leaves may be effective in preventing cancerous processes in the human body. However, the results presented are also fully questionable, as the authors of the experiment did not provide the exact concentrations of the isolated bioactive compounds from acerola, which were used to determine their cytotoxicity against the cancer cell lines used [52].

It has been observed that the substances contained in the fruit of acerola can counteract the phenomenon of photoaging of the skin, that is, have a protective effect in the process of accelerated skin aging occurring due to sunlight, mainly UVA and UVB radiation [53].

Interesting results were provided by clinical experiments using tewametric TEWL (transepidermal water loss) tests determining the level of reduction of epidermal water loss, which were carried out on 55 healthy individuals aged 45–60 years who took acerola-based products orally. As a result of the experiment, significant improvements were shown in the maintenance of skin hydration, collagen levels, the amount of sebum production, improved elasticity and the values of the skin’s L* and a* (brightness L* and color coordinate a* in the CIELAB system) indices, as manifested by a significant brightening of the skin compared to baseline values [53]. On the other hand, in an experiment on animals (mouse model) whose skin was subjected to UVA + UVB irradiation and who took acerola-based products orally, it was shown that the use of these preparations causes a significant reduction in the level of malondialdehyde (MDA) and tyrosinase activity (the enzyme that catalyzes the conversion of tyrosine into melanin) in the skin. At the same time, there was a significant increase in the level of total antioxidant activity in the skin, expressed by increased activity of the antioxidant enzymes superoxide dismutase (SOD) and glutathione peroxidase (GPx), and increased levels of reduced gluathion (GSH) in the skin [53]. For this reason, it is indicated that the consumption of nutraceuticals derived from acerola or the topical application of cosmetics enriched with acerola extract may be a promising strategy for the prevention and treatment of skin cancer, mainly through antioxidant and tyrosinase-reducing activity (maintenance of optimal melanin levels in the skin) [53].

For the described study, healthy volunteers aged 45–60 with skin type III and IV (which is characterized by moderate susceptibility to sunburn) according to the Fitzpatrick scale were recruited. The study did not involve volunteers who represent skin type II, which is easily sunburned [53].

With global warming, there is an increased amount of solar radiation reaching the Earth, mainly during the summer, including in countries in temperate climates. The consequence of these changes is an increase in the incidence of sunburn on the skin and an increased incidence of melanoma in humans as a result of increased sun exposure [54]. Therefore, including volunteers representing type II skin would also be important for a comprehensive understanding of the possibility of counteracting risks to the health and condition of the skin of people living today under conditions of increased daily total solar radiation.

The results show that acerola extract has high cytotoxic activity against HeLa cancer cell lines. An important finding was the confirmation of an inhibitory effect against the function of the Pgp protein in cancer cells. Pgp protein (P-glycoprotein) is a transport protein that is a protein of so-called multidrug resistance, belonging to the ABC (ATP-binding cassette family) of proteins. Its function is to remove hydrophobic substances (such as drugs) from the cytoplasmic part to the outer part of the lipid bilayer of the cell membrane, from where these substances then diffuse into the extracellular space. Pgp glycoproteins can also act as hydrophobic vacuum cleaners, which remove hydrophobic compounds (e.g., drugs) from the lipid layer of the cell membrane to the outside of the cell [55].

This provides the rationale that acerola-based therapeutic preparations can be used as an adjunct to therapy in situations where there is high resistance to cytostatics which is one of the main reasons for the failure of anticancer therapies in the treatment of cancers of the liver, kidney, pancreas, intestine, adrenal cortex and breast, as well as small cell lung cancer, acute and chronic myeloid leukemia or chronic lymphocytic leukemia [56]. Therefore, due to its unique antioxidant properties and high cytotoxic activity, acerola is attributed with possible applications in chemotherapy and cancer prevention [56].

A study has shown that acerola extract may be important in inhibiting cancer cell proliferation and suppressing the activity of the Ras signaling pathway during the early stages of lung cancer development. It was observed that the administration of acerola to animals (mice) at a dose of 70 mg*kg^−1^ body weight significantly inhibited the increase in the level of proliferating nuclear cell antigen and ornithine decarboxylase activity at the stage of promoting tumor transformation (carcinogenesis), when the initiation of uncontrolled divisions, the volumetric growth of tumors and the development of a network of blood vessels in the tumor (angiogenesis) occur [57,58].

The results presented here were the result of an in vivo experiment (animal model), in which only two doses of the test acerola preparation were administered to animals, which differed significantly in amount (70 mg/kg body weight and 700 mg/kg body weight) [57]. The use of a wider range of doses of the test acerola preparation would help determine the optimal amount of acerola preparation that would show effective cytotoxic effects. Perhaps the tested acerola preparation should be administered to the tested groups of animals in graded doses, which would make it possible to determine more precisely the relationship between the applied dose and the response of the tested organisms to the specific concentration of the tested acerola-derived bioactive substances.

Ascorbic acid is one of the most important bioactive substances present in acerola fruit [33]. Current in vitro and in vivo studies indicate that this antioxidant may have anticancer effects, including against liver cancer (inhibits tumor growth in vivo). It has been confirmed that ascorbic acid inhibits the viability of both liver cancer cells and selectively inhibits the development of cancer stem cells (CSC) in the liver, which play a key role in the metastasis of cancer cells from the liver to other organs [59].

Unlike most animals, humans cannot synthesize ascorbic acid due to the lack of L-gulonolactone oxidase (GLO), which catalyzes the last stage of vitamin C biosynthesis (from D-glucose), which involves the oxidation of L-gulonolactone to ascorbic acid [60].

Therefore, the recommended daily allowance (RDA) of ascorbic acid for adults (>19 years) has been established, which is 90 mg/day for men and 75 mg/day for women [60]. Research shows that consuming even just three acerola fruits a day can meet the daily requirement for vitamin C for an adult [61].

The main idea behind anti-inflammatory diets is to increase the use of ingredients with anti-inflammatory potential while limiting ingredients or products with pro-inflammatory effects. Knowledge of the anti-inflammatory properties of various acerola-derived raw materials and the areas of their effects in the body may be helpful in formulating an effective anti-inflammatory diet. The results presented in this paper indicate that the consideration of acerola in the formulation of foods with anti-inflammatory and anti-cancer properties should be combined with the use of other functional food raw materials, such as green tea (*Camellia sinensis* (L.)), whose components may act synergistically with each other. It is indicated that one of the components that may be highly synergistic with the substances contained in acerola are omega-3 polyunsaturated fatty acids, which are also attributed to a strong anti-inflammatory effect. Research also indicates that acerola is then an effective anti-inflammatory ingredient when present in foods that are both high in fibre and low on the glycaemic index.

## 11. Conclusions

The results presented in this paper indicate that acerola (*Malpighia emarginata*) has high anti-inflammatory and anti-tumour potential. Thus, acerola may be an important component of an anti-inflammatory diet to counteract excessive inflammation in the body or to alleviate already existing chronic inflammatory conditions. Acerola also exhibits anticancer activity, which has been confirmed for a number of groups of substances contained in this fruit, such as anthocyanins, polyphenolicacids and flavonoids.

Ongoing research into the biochemical properties and effects of acerola on the body will probably result in new functional food and supplement products based on the edible parts of acerola, such as pomace, seeds and leaves, in the future. This will provide consumers of different age groups, and therefore with different health problems, with products that are an important part of nutritional prevention.

## Figures and Tables

**Table 1:** Bioactive compounds of acerola fruit.

Bioactive Compounds	*Malpighia emarginata*/*Malpighia glabra*	Reference
ascorbic acid	1.18 to 2.43 g*100 g^−1^ FW (pomace)	[5]
	34 mg*100 g^−1^ FW (leaves)	[5]
β-carotene	5.84 mg*g^−1^ DM	[11]
polyphenoliccompounds	378.69 to 444.05 mg*100 g^−1^ DM	[11]
naringenin	478 μg*g^−1^ DM	[11]
cyanidin-3-rhamnoside	149–682 µg*g^−1^ FW	[5]
ferulicacid	40.96 μg*g^−1^ DM	[11]
*p*-coumaricacid	42.25 μg*g^−1^ DM	[11]
catechins	15.66 μg*g^−1^ DM	[11]
epicatechin	15.95 μg*g^−1^ DM	[11]
rutoside	36.22 μg*g^−1^ DM	[11]
total anthocyanins	19.43 mg/100 g FW	[5]
tartaricacid	1116 mg*100 g^−1^ DM	[11]
succinicacid	119 mg*100 g^−1^ DM	[11]
fructose	29.8 mg*g^−1^ DM	[12]
glucose	48.55 mg*g^−1^ DM	[12]
Antioxidant activity (EC50 as the concentration for a 50% reduction of DPPH radicals)	38.17 μg/mL (pulp)	[2]

FW—fresh weight, DM—dry matter.

**Table 2:** Human health-promoting activities of acerola fruit.

Experimental Model	The Impact on the Human Organism	Reference
In vivo	anti-atherosclerotic effect	[14]
	estrogen-like effect	[14]
	anti-inflammatory effects	[13]
	supporting the process of weight loss	[15]
	protection against lipid disorders	[15]
	increase in catalase activity	[16]
	reduction of subcutaneous fat	[17]
	improving the microbiological balance in the intestines	[18]
	gastro-protective effect (stimulation of the renewal of intestinal epithelial cells)	[19]
	inhibition of lipid peroxidation in gastric and intestinal tissues	[19]
	detoxification effect	[16]
	increasing the concentration of glutathione (GSH)	[19]
	regeneration of the gastric mucosa	[19]
	stimulation of androgen synthesis	[16]
	increase in the number of muscle fibers (hyperplasia) and enlargement of the volume of muscle fibers (hypertrophy)	[16]
	antitumor effect; reduction of the viability of breast cancer cells (MCF-7) and colon (HCT-116 cell line)	[16]
In vitro	reduction of oxidative stress in macrophages	[20]
	reduction of the secretion of the pro-inflammatory cytokines IL-1β, IL-6 and TNF-α	[20]
	increasing the secretion of anti-inflammatory cytokines such as IL-10 and TGF-β by M2 macrophage cells	[20]

## Data Availability

No new data were created.
